# The Frequency of *SMN1, SMN2* Copy Numbers in 246 Turkish Cases Analyzed with MLPA Method

**DOI:** 10.1055/s-0043-1770055

**Published:** 2023-06-16

**Authors:** Sinem Yalcintepe, Yasemin Karal, Selma Demir, Emine Ikbal Atli, Engin Atli, Damla Eker, Cisem Mail, Drenushe Zhuri, Hazal Sezginer Guler, Hakan Gurkan

**Affiliations:** 1 Department of Medical Genetics, Faculty of Medicine, Trakya University, Edirne, Turkey; 2 Department of Pediatric Neurology, Faculty of Medicine, Trakya University, Edirne, Turkey

**Keywords:** spinal muscular atrophy, SMN1, SMN2, copy number, consanguinity

## Abstract

This study aimed to define the copy numbers of *SMN1* and *SMN2* genes and the diagnosis rate and carrier frequency of spinal muscular atrophy (SMA) in the Thrace region of Turkey. In this study, the frequency of deletions in exons 7 and 8 in the *SMN1* gene and *SMN2* copy numbers were investigated. A total of 133 cases with the preliminary diagnosis of SMA and 113 cases with the suspicion of being an SMA carrier from independent families were analyzed by multiplex ligation-dependent probe amplification method for *SMN1* and *SMN2* gene copy numbers.

*SMN1* homozygous deletions were detected in 34 patients (25.5%) of 133 cases with the suspicion of SMA. Cases diagnosed with SMA type I was 41.17% (14/34), 29.4% (10/34) with type II, 26.4% (9/34) with type III, and 2.94% (1/34) with type IV. The SMA carrier rate was 46.01% in 113 cases. In 34 SMA cases, *SMN2* copy numbers were: two copies – 28 cases (82.3%), three copies – 6 cases (17.6%). *SMN2* homozygous deletions were detected in 15% (17/113) of carrier analysis cases. The consanguinity rate of the parents was 23.5% in SMA diagnosed cases. In this study, we had a 25.5% of SMA diagnosis rate and 46% SMA carrier frequency. The current study also showed the relatively low consanguinity rate of the Thrace region, with 23.5% according to the east of Turkey.

## Introduction

Spinal muscular atrophy (SMA) is a group of hereditary neuromuscular diseases with autosomal recessive, X-linked recessive, or autosomal dominant inheritance. 1 This disorder is characterized by muscle weakness and atrophy as a result of progressive degeneration and loss of anterior horn cells in the spinal cord and the brainstem nuclei. 2 The incidence is estimated approximately 1 in 11,000 births, the average global carrier frequency is 1/50. 3 4

*SMN1* gene is mapped on chromosome 5q13.2. The role of the *SMN1* gene is to produce survival motor neuron (SMN) protein, which is highly expressed in the spinal cord and is essential for motor neuron survival. 5 The *SMN* gene has two forms in humans, telomeric ( *SMN1* ) and centromeric *SMN2* . *SMN2* is nearly identical in genomic sequence to *SMN1* , and there are only five nucleotides different. 6 The critical sequence difference between the two genes is a single nucleotide in exon 7, the C-to-T nucleotide difference of the *SMN2* gene creates an exonic splicing suppressor that leads to a skipping of exon 7 during pre-messenger ribonucleic acid splicing. 7 This results in *SMN2* producing a truncated, nonfunctional, and rapidly degrading SMN protein. The disease begins with denervation due to decreased SMN level in medulla spinalis anterior horn α-motor neurons. The lower extremities are more affected than the upper extremities, the proximal muscles, than the distal muscles. 8 Complications such as respiratory and nutritional problems negatively affect the patients' prognosis.

Patients with SMA usually have normal or high intelligence levels. 9 The most common form, autosomal recessive proximal SMA, is caused by pathogenic variations of the *SMN1* gene. 10 Autosomal recessive proximal SMA has been subdivided into five main types (type zero, I, II, III, and IV) based on the age of onset and maximum motor function achieved ( Table 1 ) in untreated patients (3). SMA type I (OMIM #253300) (Werdnig–Hoffmann disease) is the most frequent subtype, and is characterized by inability to independently sit, rapidly progressive motor, respiratory, and bulbar deterioration, and > 90% mortality by 2 years of age. 11 Children with SMA type II (OMIM #253550) can sit independently but never walk alone, and many of the patients survive into adulthood. 12 Patients with SMA type III (Kugelberg–Welander disease) (OMIM #253400) can walk at some point in their lifetime, some cases may continue to walk, some may not. 3 Type IV is the mildest form with adult onset. 10 The most severe type SMA is type zero. Infants with SMA type zero have severe respiratory failure, rarely survive past the age of 6 months, and may have a history of decreased in utero movements, joint contractures, and atrial septal defects at the perinatal period. 13

**Table 1:** Clinical characteristics of the spinal muscular atrophy (SMA) types a

Phenotype	Age of onset	Life span	Motor milestones	Other findings
SMA type 0 (prenatal SMA, congenital SMA)	Prenatal	A few weeks, < 6 mo	None achieved	Severe neonatal hypotonia Severe weakness Areflexia Respiratory failure at birth Facial diplegia ↓ fetal movements Atrial septal defects Arthrogryposis
SMA type I (Werdnig–Hoffman disease, infantile SMA)	< 6 mo	Median survival 8–10 mo	Some head control, sit w/support only	Loss of head control Mild joint contractures Normal or minimal facial weakness Variable suck and swallow difficulties
SMA type II (Dubowitz type)	6–18 mo	70% alive at age 25 y	Independent sitting when placed	Developmental delay w/loss of motor skills ↓ or absent deep tendon reflexes Proximal muscle weakness Postural tremor of fingers
SMA type III (Kugelberg– Welander disease, juvenile SMA)	> 18 mo	Normal	Independent ambulation	Proximal muscle weakness (i.e., difficulty w/stairs, running) Loss of motor skills Fatigue Postural tremor of fingers Loss of patellar reflexes
SMA type IV (adult type SMA)	Adulthood	Normal	Normal	Fatigue Proximal muscle weakness

a Prior TW, Leach ME, Finanger E. Spinal Muscular Atrophy. 2000 Feb 24 [Updated 2019 Nov 14]. In: Adam MP, Ardinger HH, Pagon RA, et al., editors. GeneReviews® [Internet]. Seattle, WA: University of Washington, Seattle; 1993–2020. Available from: https://www.ncbi.nlm.nih.gov/books/NBK1352/ .

In more than 95% of patients with SMA, exon 7 of the *SMN1* gene is mutated by deletion or gene conversion. 14 Multiple different mechanisms are responsible for the loss of the *SMN1* gene. The first mechanism is de novo mutations that occur during paternal meiosis due to imbalanced crossing-over caused by duplication and inversion. The second mechanism is converting the *SMN1* gene to the *SMN2* gene, as the copy number of *SMN2* increases in such patients, the severity of the disease decreases. 15 But there may be some exceptions; for example, while there is type I cases with two SMN2 copy numbers, there are also some type III cases with two SMN2 copy numbers.

SMA newborn screening in Turkey was first started recently, May 2022. The aim of this study was to investigate exon 7 and 8 deletion frequencies in the *SMN1* gene in 133 patients with a preliminary diagnosis of SMA and 113 cases for SMA carrier analysis in the Thrace region from Turkey.

## Material and Methods

### Study Group

This study was a retrospective design of 8.5 years' (January 2012–June 2020) follow-up, including 133 individuals with the suspicion of SMA and 113 unrelated SMA carrier analysis cases from independent families. Written informed consent forms were obtained from the cases or parents. This study is approved by the Ethical Committee of our university with the number 2020/330 and performed in accordance with the principles of the Declaration of Helsinki.

### Inclusion Criterias of the Cases

A total of 246 cases were included in this study. A total of 133 individuals with the suspicion of SMA were included with SMA clinical findings (hypotonia, muscle weakness and atrophy, decreased or absent reflexes, and twitching of muscle fibers, fasciculations). A total of 113 unrelated cases from independent families were included for SMA carrier analysis. SMA carrier analysis was done for testing before pregnancy, anxiety for being an SMA carrier, or having an SMA diagnosed or SMA carrier relative.

### *SMN1* / *SMN2* Analysis

Two milliliters of peripheral blood was taken from each proband and/or his/her parents into an ethylenediaminetetraacetic acid anticoagulation tube. Genomic deoxyribonucleic acid (DNA) was extracted using EZ1 DNA Investigator Kit (Qiagen, Hilden, Germany). Primary quality control of the isolated DNA samples was performed using NanoDrop (Thermo Fisher Scientific, Waltham, Massachusetts, United States), and samples having A260/280 values between 1.8 and 2.0 were included in the study.

**Fig. 1:**
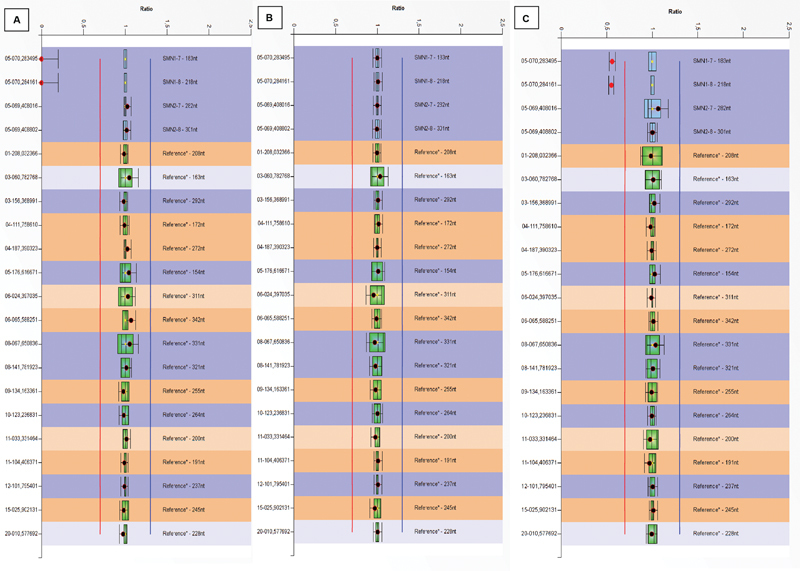
Multiplex ligation-dependent probe amplification (MLPA) result screens of ( **A** ) SMN1 gene exon 7 and exon 8 homozygous deletion of an spinal muscular atrophy (SMA) diagnosed case, ( **B** ) no deletion of SMN1 gene, and ( **C** ) heterozygous deletion of SMN1 exon 7 and exon 8 of an SMA carrier case.

The extracted genomic DNA was diluted to 40 ng/μL, and 5 μL was taken and used for multiplex ligation-dependent probe amplification (MLPA) analysis. *SMN1* and *SMN2* gene copy numbers were detected using MLPA with the SALSA P060-B2 SMA Kit (MRC Holland) according to the manufacturer's protocol. Polymerase chain reaction products were analyzed on the ABI 3130 Genetic Analyzer (Applied Biosystems), and data were analyzed by Coffalyzer software (MRC Holland). Ratio 0.5 indicated heterozygous deletion, while ratio 0 indicated homozygous deletion ( Fig. 1 ). Ratios < 0.7, 0.7 < ratio < 1.3, 1.3 < ratio < 1.7, and 1.7 < ratio < 2.3 indicated one, two, three, and four gene copies, respectively.

## Results

We analyzed 246 cases, 133 with the suspicion of SMA and 113 for SMA carrier analysis. Using MLPA, we found that approximately 25.56% (34/133) of patients had homozygous deletions for *SMN1* . Three of these 34 patients had only *SMN1* exon 7 deletion, and other 31 patients had both exon 7 and exon 8 deletions. Among these SMA patients, 41.17% (14/34) of patients have been diagnosed with SMA type I, 29.4% (10/34) with type II, 26.4% (9/34) with type III, and 2.94% (1/34) with type IV ( Table 2 ). In SMA carrier testing, 52 cases (46.01%) of 113 had *SMN1* exon 7 and exon 8 heterozygous deletions.

**Table 2:** SMA types and *SMN2* copy numbers of *SMN1* homozygous deletion detected cases in our study

SMA types	*SMN1* homozygous deletion (34/133) (25.56%)	*SMN2* copy number
SMA type 0	−	−
SMA type I	41.17% (14/34)	2 (12 cases), 3 (1 case)
SMA type II	32.35% (11/34)	2 (10 cases), 3 (2 cases)
SMA type III	23.52% (8/34)	2 (6 cases), 3 (2 cases)
SMA type IV	2.94% (1/34)	3 (1 case)

Abbreviations: SMA, spinal muscular atrophy; SMN1, survival of motor neuron 1; SMN2, survival of motor neuron 2.

In 34 SMA cases, the distribution of *SMN2* copy number was as follows: two copies – 28 cases (82.3%), three copies – 6 cases (17.6%). *SMN2* homozygous deletions were detected in 15% (17/113) carrier analysis cases.

Thirty-four patients diagnosed as SMA had different clinical features for testing: hypotonia, neuromotor growth retardation, fasciculations, walking difficulties, muscle weakness, and one SMA type III case with increased creatine kinase level (472 U/L). A 49-year-old case, who has only progressive walking difficulty, was diagnosed as SMA type IV with three *SMN2* copy numbers. Two cases were brothers in the current study; a 14-year-old case was diagnosed with SMA type III in our study. Parents were first-degree cousins, and therefore his brothers were tested for SMA, too. And the 3-year-old brother, with no SMA clinical findings, was diagnosed with SMA after testing.

The consanguinity rate of the parents of SMA-diagnosed cases in our study was 23.5%.

## Discussion

In the current study, *SMN1* and *SMN2* gene deletions were examined in patients with the suspicion of SMA by MLPA method, and 25.5% of the patients had *SMN1* exon 7 and 8 homozygous deletions. In 52/113 (46.01%) cases, *SMN1* gene exons 7 and 8 heterozygous deletions were detected. Italian, Spanish, and English/Scottish populations were reported with at least one *SMN1* /2 copy differing between 15 and 20%. 16 In China, the overall carrier rate of SMA was reported as 2%. 17 It seems like the carrier prevalence is higher in Turkey. In a study from Turkey, in positive samples, 88.13% of cases had *SMN1* exons 7 and 8 homozygous deletions, and 54.5% had heterozygous deletions of *SMN1* . 18 Our study has a high rate of SMA as well as in Turkey, possibly due to the high frequency of consanguineous marriages in our country.

Copy numbers of the *SMN2* gene in SMA types II and III were reported to be equal to or greater than three in the first genotype-phenotype correlation studies. 19 20 Mildly clinical features of SMA were reported as patients have higher *SMN2* gene copies, probably due to conversion from the *SMN1* gene. 21 Decreased *SMN2* copy number is reported as correlated with the *SMN1* copy number in the general population. 22 The clinical features that should be pointed out in our study vary when patients have the same *SMN2* copy numbers. Only two patients had partial head control among the 12 cases with SMA type I with two copies of the *SMN2* gene. Half of the patients with SMA type II with two copies of the *SMN2* gene had walking difficulties. In the case of a 10-year-old patient with homozygous deletion of *SMN1* and 3 copies of *SMN2* gene with SMA type III diagnosis, supporting studies reporting increased copy number of *SMN2* may compensate for the lack of *SMN1* gene and cause to a milder SMA phenotype. In another case, 49 years old, presenting with only progressive walking difficulty, was diagnosed as SMA type IV with three *SMN2* copy numbers.

A 14 years old case with walking difficulty and muscle weakness was first diagnosed with SMA type III, and his other three brothers were also tested for SMA. Only the 3-year-old case had *SMN1* exon 7 and 8 homozygous deletion like his 14-year-old brother. At age 3, he had no clinical findings related to SMA type III. In a study from Turkey, molecular genetic characterization was analyzed in 24 SMA type III patients. The same study reported that all cases had symmetrical proximal-distal weakness and lower motor neuron involvement with a mean age of 29 years for male and 30 years for female patients. 23 The mean age of SMA type III patients in our study was 23 with the youngest case was 3 years old.

The rate of consanguineous marriages in our study was 23.5%; an SMA study from Iran reported 97% consanguinity, 24 68% consanguinity was reported in a study from Turkey, 18 a prenatal diagnosis study from Turkey reported consanguinity in 21 of 63 families 25 because autosomal recessive diseases are a common result of consanguinity. The rate of consanguinity in our study is at a lower rate for our country. This may be because our cases are mostly from the Thrace region, northwest of Turkey, where consanguineous marriages are at a lower rate. Our consanguinity rate shows the importance of regional differences of Turkey.

According to the literature, the detection rate of SMA diagnosis in our study (25.5%) is relatively low. 18 Our cases were included in the study for having clinical findings like hypotonia, neuromotor growth retardation, walking difficulties, muscle weakness and atrophy, and decreased or absent reflexes. SMA is an excluding diagnosis for these patients in the algorithm. If the case had not been diagnosed as SMA, advanced tests are provided. Our detection rate may be low because all hypotonia cases are tested for SMA. SMA carrier rate is high in our country as high as consanguineous marriages. Forty-six percent of our carrier analysis cases were SMA carriers in the current study.

There are some limitations of this study. One hundred and thirty-three individuals with the suspicion of SMA were included in our research, and only 34 were diagnosed as SMA after the molecular genetics analysis. Larger samples would be more informative about the prevalence and genotype-phenotype correlation of SMA. And also we could not analyze *SMN1* sequencing to detect point mutations or small insertions/deletions. Another limitation was that electroneuromyography or creatinine kinase levels of the individuals could not be reported in this article.

Autosomal recessively inherited diseases such as SMA are frequently seen in countries with high consanguinity marriages like Turkey. Since SMA carriage rates are high in our country, it should be offered to all couples with or without an SMA diagnosed child in their family before pregnancy. When the couple is determined to be carriers, genetic counseling, prenatal, or preimplantation diagnostic testing options may be offered. Social support and genetic counseling should be available for the population to decrease the rates of these diseases. SMA is at a 25% risk of recurrence for carrier couples and a disease can be diagnosed with prenatal diagnosis; it is essential to establish the molecular genetic diagnosis of affected patients. 26 Parents should be informed about carrier screening, newborn screening, prenatal diagnosis, and preimplantation genetic diagnosis.

Due to the fact that some neurological diseases have similar clinical features, it is difficult to make a definitive clinical diagnosis of the disease. The diagnosis is tried to be based on clinical examination and electrophysiological criteria, but sometimes differential diagnosis cannot be made due to some variant types of SMA. SMA diagnosis can be made more easily today, but similar clinical conditions should be considered. The discovery of new treatment methods has been promising for SMA patients.

The current study presenting 246 cases, 133 with the suspicion of SMA and 113 for SMA carrier analysis, SMA genotypes and phenotypes from the Thrace region of Turkey make a different contribution to the literature with a 25.5% SMA diagnosis rate and 23.5% consanguinity rate. The west of Turkey differs from the east region with consanguinity rates. In the current study, 41.17% (14/34) of patients have been diagnosed with SMA type I, 29.4% (10/34) with type II, 26.4% (9/34) with type III, and 2.94% (1/34) with type IV, and 46% of 113 SMA carrier analysis cases had been diagnosed as SMA carriers. Turkey has a high SMA carrier frequency, and with the screening program in our country, we hope that SMA patient frequency will decrease.
